# Re-assessment of schistosomiasis in nine endemic districts with cluster sampling in Sierra Leone

**DOI:** 10.3389/fpubh.2024.1415486

**Published:** 2024-06-12

**Authors:** Ibrahim Kargbo-Labour, Victoria Redwood-Sawyerr, Abdulai Conteh, Elisabeth Chop, Mohamed S. Bah, Abdulai Koroma, Unidiatu Kabia, Luke Klein, Sugandh Juneja, Patricia Houck, Steven Reid, Darin Evans, Angela M. Weaver, Anna E. Phillips, Yaobi Zhang

**Affiliations:** ^1^National Neglected Tropical Disease Control Program, Ministry of Health and Sanitation, Freetown, Sierra Leone; ^2^Helen Keller International, Freetown, Sierra Leone; ^3^Helen Keller International, New York, NY, United States; ^4^FHI 360, Washington, DC, United States; ^5^United States Agency for International Development, Washington, DC, United States

**Keywords:** schistosomiasis, *Schistosoma mansoni*, *Schistosoma haematobium*, impact assessment, cluster sampling, Sierra Leone

## Abstract

**Background:**

Baseline mapping showed that schistosomiasis was highly/moderately endemic in nine districts in Sierra Leone. Mass drug administration (MDA) with praziquantel started in 2009, and after multiple rounds of treatment, an impact assessment was conducted in 2016 followed by a second re-assessment in 2022 using cluster sampling to provide more granular data for refining chiefdom (sub-district) treatment strategies.

**Methods:**

On average, 20 rural villages were systematically selected per district by probability proportional to population size across the nine districts. Surveys were conducted in schools, and 24 school children aged between 5 and 14 years were randomly selected, with an equal number of boys and girls. One stool sample and one urine sample were collected per child. Two Kato-Katz slides were examined per stool for *Schistosoma mansoni* infection. Hemastix strips were used as a proxy for *S. haematobium* infection with urine filtration used for egg counts on hematuria-positive samples.

**Results:**

In total, 4,736 stool samples and 4,618 urine samples were examined across 200 schools in 125 chiefdoms. Overall, the prevalence of *S. mansoni* was 16.3% (95% CI: 15.3–17.4%), while the overall prevalence of *S. haematobium* was 2.0% (95% CI: 1.6–2.4%) by hematuria. The prevalence of heavy infections for *S. mansoni* and *S. haematobium* was 1.5% (95% CI: 1.1–1.9%) and 0.02% (95% CI: 0.0–0.14%), respectively. Among 125 chiefdoms surveyed, the overall schistosomiasis prevalence was <10% in 65 chiefdoms, 10–49.9% in 47 chiefdoms, and ≥ 50% in 13 chiefdoms. There was a mixed relationship between schistosomiasis in school children and WASH access in schools.

**Conclusion:**

Sierra Leone has made significant progress in reducing schistosomiasis prevalence across the country after a decade of MDA intervention. However, high prevalence remains in some hotspot chiefdoms. The next steps are for the national program to investigate and address any potential issues such as low coverage or poor knowledge of schistosomiasis risk behaviors and, where appropriate, consider broadening to community-wide treatment in hotspot chiefdoms or communities.

## 1 Introduction

Human schistosomiasis is caused by infection with the trematode *Schistosoma* spp., predominantly *S. haematobium*, *S. mansoni,* and *S. japonicum*, with the former causing urogenital schistosomiasis and the latter two causing intestinal schistosomiasis (1). It is one of the most common neglected tropical diseases currently targeted for elimination as a public health problem (2). In 2021, an estimated 251.4 million people (136 million school-aged children (SAC) and 115.4 million adults) in 51 tropical and subtropical countries required regular treatment, with an overwhelming 90.6% of these people located in 41 countries in Africa (3). The predominant strategy for schistosomiasis control recommended by the World Health Organization (WHO) has been preventive chemotherapy, through which at-risk populations are given regular treatment with praziquantel (4, 5). Endemic districts are classified as low (<10% prevalence), moderate (10 to <50% prevalence), or high (≥50% prevalence) endemicity during baseline mapping, following which associated treatment frequencies result in mass drug administration (MDA), typically implemented at district level among school-aged children (4, 6). Over the last two decades, with the availability of international funding, praziquantel donations, and internal government support, preventive chemotherapy for schistosomiasis has significantly scaled up in many countries, most notably in sub-Saharan Africa, resulting in considerable reductions in schistosomiasis prevalence (7). In 2022, the WHO published new recommendations advocating for treatment of all age groups above 2 years of age in areas where prevalence was found to be greater than 10% (5). However, due to the limited availability of donated praziquantel, there are not enough drugs to treat all at-risk groups, and there have been calls to shift mass treatment from district level to sub-district/community level. To facilitate this transition, more granular data at the sub-district/community level are required.

In Sierra Leone, based on mapping conducted in 2008–2009 and previous WHO recommendations (4, 8–10), schistosomiasis endemic districts were classified as having low prevalence in five coastal districts, moderate prevalence in four districts, and high prevalence in five districts. Two districts, Bonthe and Western Area Urban, were found to be non-endemic for schistosomiasis. *S. mansoni* is the predominant schistosome species in nine districts (8, 9), and *S. haematobium* is endemic mainly in three districts (Bo, Kono, and Koinadugu) (10). In 2009, MDA with praziquantel was launched in six (three with high and three with moderate prevalence) endemic districts targeting only school-attending children, and it was scaled up in 2010 to include all SAC and at-risk adults (any adult living in rural areas) in the nine highly or moderately endemic districts, as per the national plan for morbidity control (11, 12). MDA was given annually in highly endemic districts and biennially in moderate districts. The five low-endemic coastal districts have received no MDA to date.

As per the WHO guidelines (6), in 2016, schistosomiasis evaluation was conducted after 5–6 years of preventive chemotherapy in all 16 districts (13). The results showed that the overall schistosomiasis prevalence decreased from 42.2% at baseline to 20.2% in 2016 in the nine highly or moderately endemic districts. Accordingly, these nine originally moderate-to-high prevalence districts were re-classified as follows: low in two districts and moderate in seven districts, with no high prevalence districts. The five low-prevalence coastal districts that had received no MDA remained low endemic, and the two non-endemic districts remained non-endemic at the 2016 assessment. Following this impact assessment, the treatment strategy shifted to targeting SAC only and implementing MDA at the chiefdom level (equivalent to a sub-district) in 2017. Chiefdoms are targeted for MDA based on 2016 prevalence data in accordance with the WHO 2011 Guidelines (6).

After completing five further rounds of MDA in 7 years, another impact assessment survey was conducted in nine districts in October–November 2022, 6 months after the last MDA, to assess the impact of treatment on schistosomiasis since the last survey conducted in 2016. This study presents the results of the impact assessment in 2022 and discusses the lessons learned, the implications of the new WHO 2022 recommendations to redefine treatment strategies and limitations of the survey.

## 2 Methods

### 2.1 Mass drug administration

Schistosomiasis MDA is conducted in Sierra Leone through a combined community- (for non-attending SAC and adults) and school-based strategy. Trained health facility workers conduct directly observed treatment. As many children in rural settings often go to school in the morning without a meal, for school-based MDA children were given food to take with the drug at the time of the MDA to both minimize the chance of side effects and maximize treatment coverage (11). A special arrangement was made by the national program with school authorities to facilitate local meal preparation, such as boiled cassava or rice mixed with sauce (locally called “wan pot”), for children on the day of MDA taking place. The distribution typically lasts for a week with mop-ups deployed if coverage targets are not met. Health facility staff record treatments in paper-based tally sheets, and at the end of each MDA coverage, data are aggregated through the country’s health pyramid (Primary Health Unit, district, regional, and national levels).

### 2.2 Selection of survey sites

A cross-sectional survey was conducted in October–November 2022, 6 months after MDA. A two-stage cluster sampling design was used to estimate prevalence at the chiefdom level, surveying 15–20 schools per district and 1–5 schools per chiefdom, according to Knowles et al. (14). Villages were selected from an exhaustive list of villages in each district according to the following steps: (1) rural villages were systematically selected using probability proportional to population size across the nine districts; (2) to ensure all chiefdoms were represented, for chiefdoms with small population sizes that were missed from the general sampling, one village was randomly selected from each; and (3) to avoid sampling bias, large urban areas were excluded from the Step 1 sampling. Instead, two communities from each large town were randomly selected. This resulted in 1–5 villages being sampled in each chiefdom relative to population size. The survey was conducted in primary schools in selected villages. When there is more than one school in one village, one school was randomly selected.

In total, 206 schools were selected in 127 chiefdoms across 9 districts. A total of 197 schools were selected from rural villages (Bo: 22, Bombali: 16, Falaba: 14, Kailahun: 27, Karene: 16, Kenema: 27, Koinadugu: 17, Kono: 29, and Tonkolili: 29) and 9 schools from large district towns (Bo city: two, Kenema city: two, Koidu city: two, Makeni city: two, and Kailahun city: one). Survey teams were unable to collect samples from six schools due to issues with authorization and access in these sites; as a result, a total of 200 schools were surveyed.

### 2.3 Selection of children in schools

In each school, 24 children aged between 5 and 14 years were randomly selected for inclusion in the survey, with equal numbers from boys and girls. In general, children were arranged into lines by sex and by grade, and two boys and two girls were randomly selected from each of six classes/grades using a sampling interval and a random starting point. There was one all-boys school in Bo district where all 24 participants were boys. In total, 4,736 SAC were sampled.

### 2.4 Diagnosis

Each selected child was given two containers, one for urine and one for stool, and asked to provide a sample for each. Duplicate Kato-Katz was used to assess *S. mansoni* infection on a single stool sample examined within 1 h of preparation. The number of eggs identified was counted and recorded for each slide and averaged across the two slides (6, 15). Urinalysis reagent strips (Hemastix, Siemens, Terrytown, US) were used as a proxy for the diagnosis of *S. haematobium*, where a urine sample was considered positive by Hemastix for *S. haematobium* infection when the test showed “trace hemolyzed,” “+,” “++,” or “+++” for microhematuria (16). All microhematuria-positive urine samples were further examined with the urine filtration method for egg counts. A volume of 10 mL of urine was filtered and examined on one slide for *S. haematobium* eggs under light microscope. A child was considered positive for *S. haematobium* infection when Hemastix test was positive for any schistosomiasis if either Hemastix and/or Kato-Katz was positive. All egg-positive slides and 10% of egg-negative slides were examined by a senior technician for quality control.

### 2.5 Data research topic and analysis

Annual MDA data were the reported data from the national program. Electronic forms on mobile tablets for survey data Research collection were designed using the Ona platform (17), to collect sociodemographic information on sex and age of each child, laboratory results of stool and urine sample examinations, and information on water, sanitation, and hygiene (WASH) facilities at each school. The global positioning system (GPS) coordinates of each surveyed school were collected using the tablets for data entry.

The results were then downloaded in an Excel spreadsheet and cleaned. All individuals with missing stool and urine samples were removed from the final dataset. The final dataset was analyzed using R (v4.3.0; R Core Team 2023). Intensity of infection was calculated using number of eggs per gram of feces (epg) for *S. mansoni* by multiplying the average egg counts from two slides by 24 and number of eggs per 10 mL of urine (eggs/10 mL) for *S. haematobium*. Intensity of infection for individual child was classified as heavy (≥400 epg), moderate (100–399 epg), or light (<100 epg) for *S. mansoni* infection and heavy (≥50 eggs/10 mL) or light (<50 eggs/10 mL) for *S. haematobium* infection. Unadjusted prevalence of infection and arithmetic mean egg counts among all children examined with 95% confidence intervals (CIs) from all SAC examined were calculated using the binomial exact test to account for the binary structure of the data. The WASH data collected were categorized as basic service, limited service, or no service using the WHO/UNICEF Joint Monitoring Program WASH in schools’ indicators (18). The chi-squared test was used to compare the prevalence between districts, sex, age, and access to WASH facilities. The GPS coordinates collected for each school were used to conduct geostatistical modeling of the predicted prevalence distribution using the kriging methods in the Geostatistical Analyst Extension of the ArcGIS version 10.8.2 (ESRI, Redlands, California, United States).

## 3 Results

### 3.1 MDA coverage

**Figure 1:**
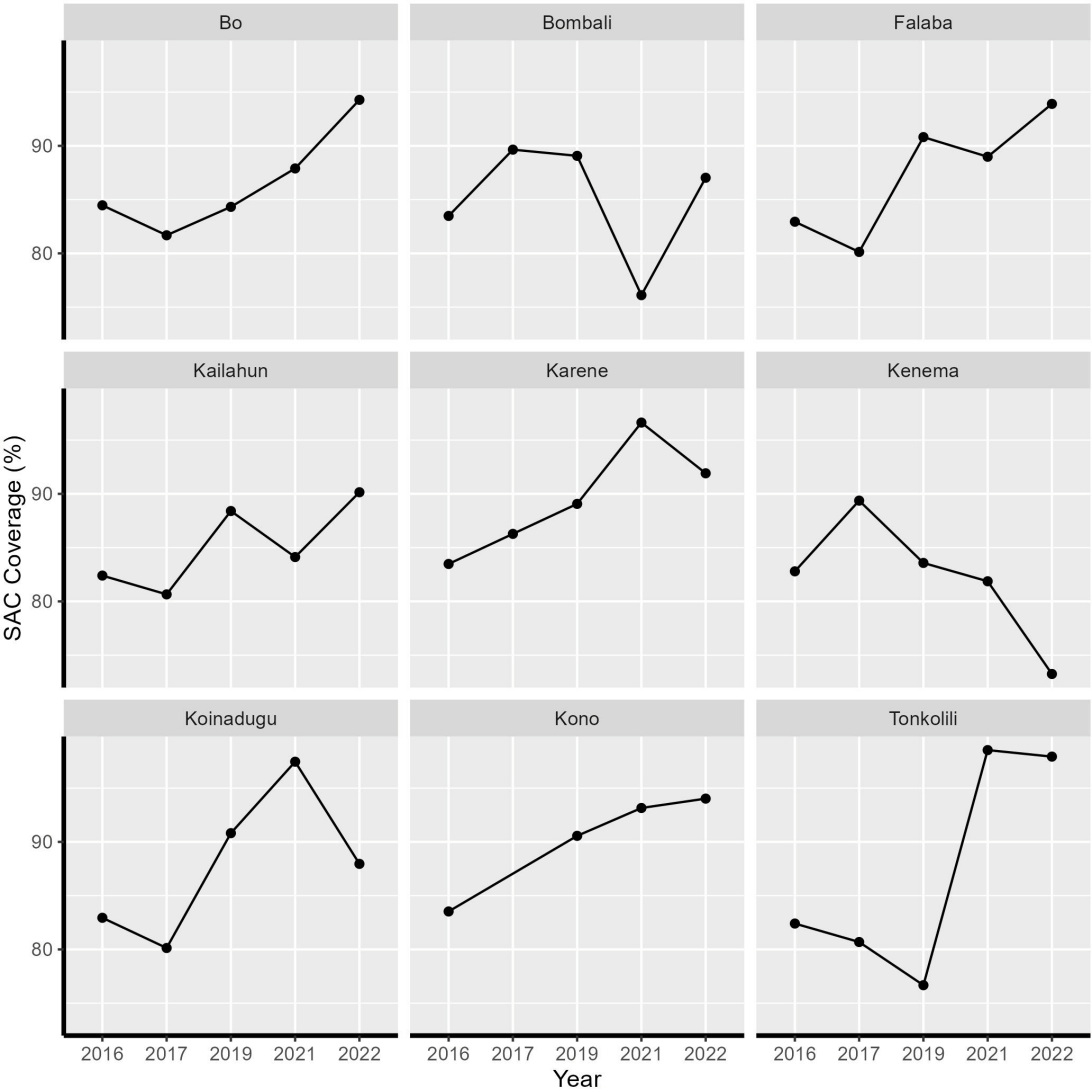
Treatment coverage of school-aged children from 2016 to 2022 in Sierra Leone by health district.

Between 2016 and 2022, Sierra Leone implemented five rounds of praziquantel MDA. Initially, both SAC and high-risk adults were targeted for treatment in 2016 and 2017, followed by a shift to exclusively target SAC in subsequent years. There was no MDA in 2018 due to change in projects. MDA resumed in 2019 but was interrupted by COVID-19 in 2020. There has been high treatment coverage across all nine districts over the five treatment rounds. All districts achieved the recommended coverage threshold of 75% for SAC, except for Kenema district in 2022, where the coverage was 73.25% (Figure 1).

### 3.2 Prevalence and distribution

**Figure 2:**
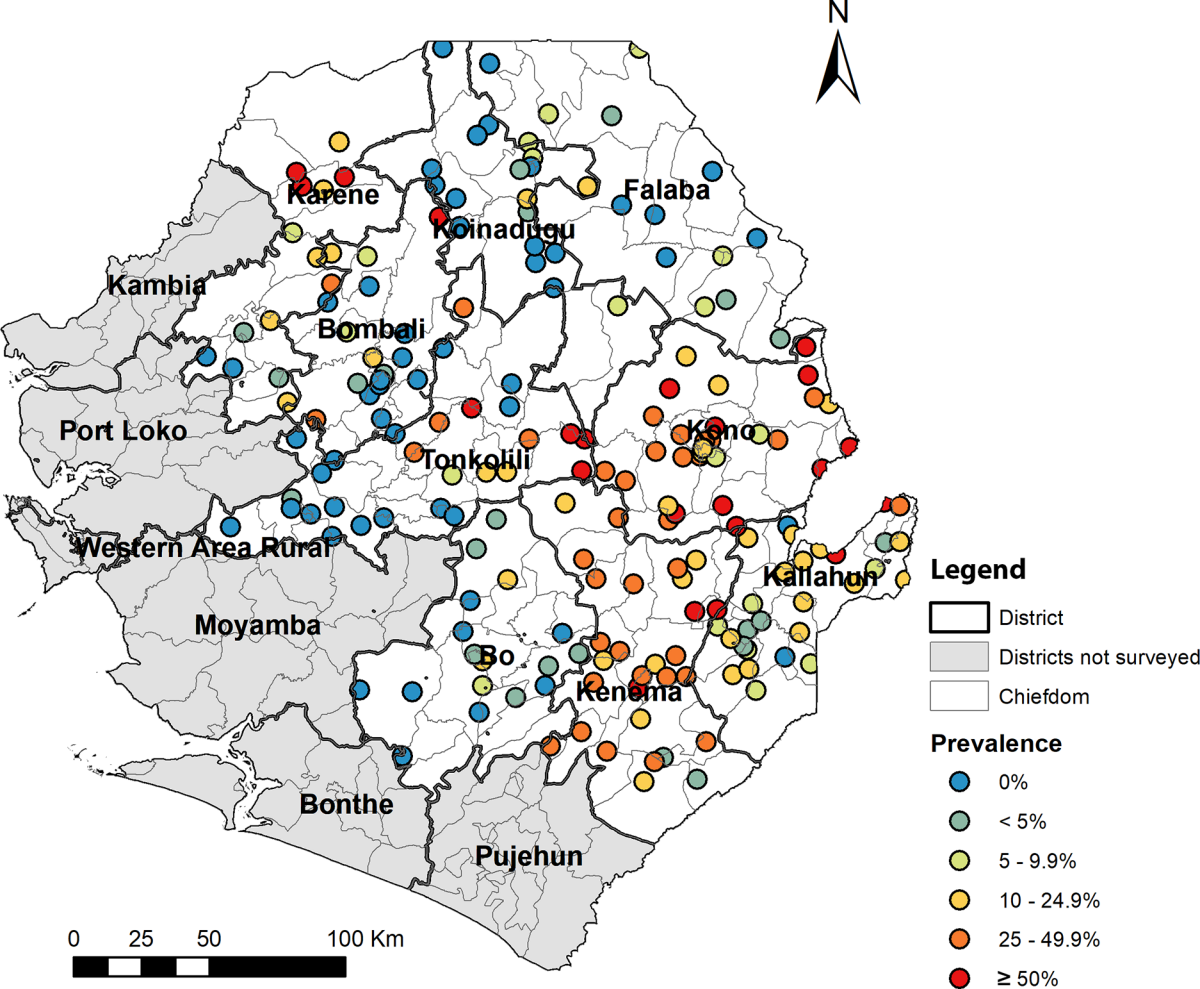
Distribution and point prevalence of any schistosomiasis in 2022.

**Figure 3:**
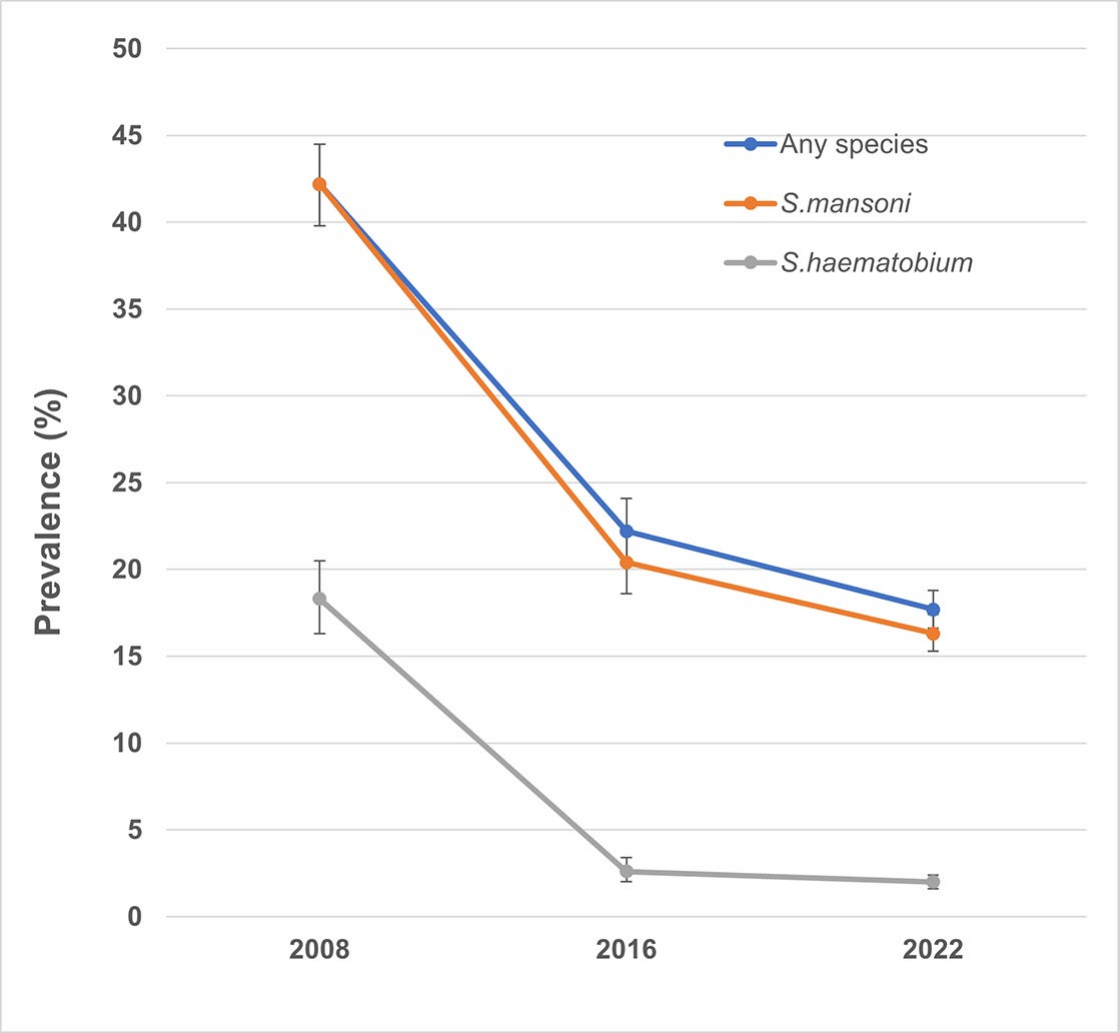
Trend of prevalence of any schistosomiasis over the years in Sierra Leone. *S. haematobium* and *S. mansoni* were evaluated separately at baseline, so the prevalence of *S. mansoni* was used as a proxy for any species infection in the figure.

In total, 4,736 children were enrolled across 200 schools in 125 chiefdoms, and 4,736 stool samples and 4,618 urine samples were collected and examined (Table 1). Overall, the prevalence of *S. mansoni* was 16.3% (95% CI: 15.3–17.4%), while the overall prevalence of *S. haematobium* by hematuria was 2.0% (95% CI: 1.6–2.4%) and 1.8% (95% CI: 1.4–2.2%) by urine filtration. Overall, the prevalence for any schistosome species was 17.7% (95% CI: 16.7–18.8%), ranging from 0 to 91.7% among 200 schools (Figure 2). Half of the 200 schools surveyed had a prevalence of greater than 10%, of which 22 schools had a prevalence of ≥50%. Figure 3 shows the trend of prevalence between 2008, 2016, and 2022. Overall, the prevalence decreased from 42.2% in 2008 to 22.2% in 2016 and to 17.7% in 2022, in which the prevalence of *S. mansoni* decreased from 42.2% in 2008 to 20.4% in 2016 and to 16.3% in 2022, while the prevalence of *S. haematobium* decreased from 18.3% in 2008 to 2.6% in 2016 and to 2.0% in 2022.

**Table 1:** Prevalence of schistosomiasis in school-aged children in 2022.

District	Number of sites surveyed	Number of school children examined with stool samples	Number of stool samples tested positive	Prevalence (%) of *S. mansoni* infection (95% CI)	Number of school children examined with urine samples	Number of urine samples tested positive by Hemastix and/or filtration^*^	Prevalence (%) of *S. haematobium* by Hemastix and/or filtration (95% CI)	Number of school children positive for any species	Prevalence (%) of any species (95% CI)
Bo	18	432	14	3.2 (1.9–5.5)	429	1	0.2 (0–1.5)	15	3.5 (2.0–5.8)
Bombali	18	418	16	3.8 (2.3–6.3)	418	32	7.7 (5.4–10.7)	41	9.8 (7.2–13.2)
Falaba	14	330	4	1.2 (0.4–3.3)	329	13	4 (2.2–6.8)	13	3.9 (2.2–6.8)
Kailahun	28	670	96	14.3 (11.8–17.3)	645	0	0 (0–0.7)	96	14.3 (11.8–17.3)
Karene	16	368	88	23.9 (19.7–28.6)	366	10	2.7 (1.4–5.1)	90	24.5 (20.2–29.2)
Kenema	29	685	180	26.3 (23.1–29.8)	629	31	4.9 (3.4–7)	201	29.3 (26.0–32.9)
Koinadugu	17	407	12	2.9 (1.6–5.2)	406	0	0 (0–1.2)	12	2.9 (1.6–5.2)
Kono	31	737	257	34.9 (31.5–38.5)	733	6	0.8 (0.3–1.9)	260	35.3 (31.8–38.8)
Tonkolili	29	689	107	15.5 (13.0–18.5)	663	7	1.1 (0.5–2.3)	111	16.1 (13.5–19.1)
Total	200	4,736	774	16.3 (15.3–17.4)	4,618	100	1.8 (1.4–2.2)	839	17.7 (16.7–18.8)

^*^There were 21 Hemastix-negative urine samples which were tested by filtration, and the results were included in the urine results.

The prevalence by district ranged from 1.2% (95% CI: 0.4–3.3%) in Falaba to 34.9% (95% CI: 31.5–38.5%) in Kono for *S. mansoni*, from 0 (95% CI: 0–0.7%) to 7.7% (95% CI: 5.4–10.7%) for *S. haematobium* for microhematuria, from 0 to 5.3% for urine filtration, and from 2.9 to 35.3% for any species infection (Table 1). Four districts (Bo, Bombali, Falaba, and Koinadugu) had low prevalence (≥1 and < 10%) and five districts (Kailahun, Karene, Kenema, Kono, and Tonkolili) had moderate (≥10 and < 50%) prevalence for *S. mansoni*. *S. haematobium* infection was primarily found in four districts (Bombali, Falaba, Karene, and Kenema).

**Figure 4:**
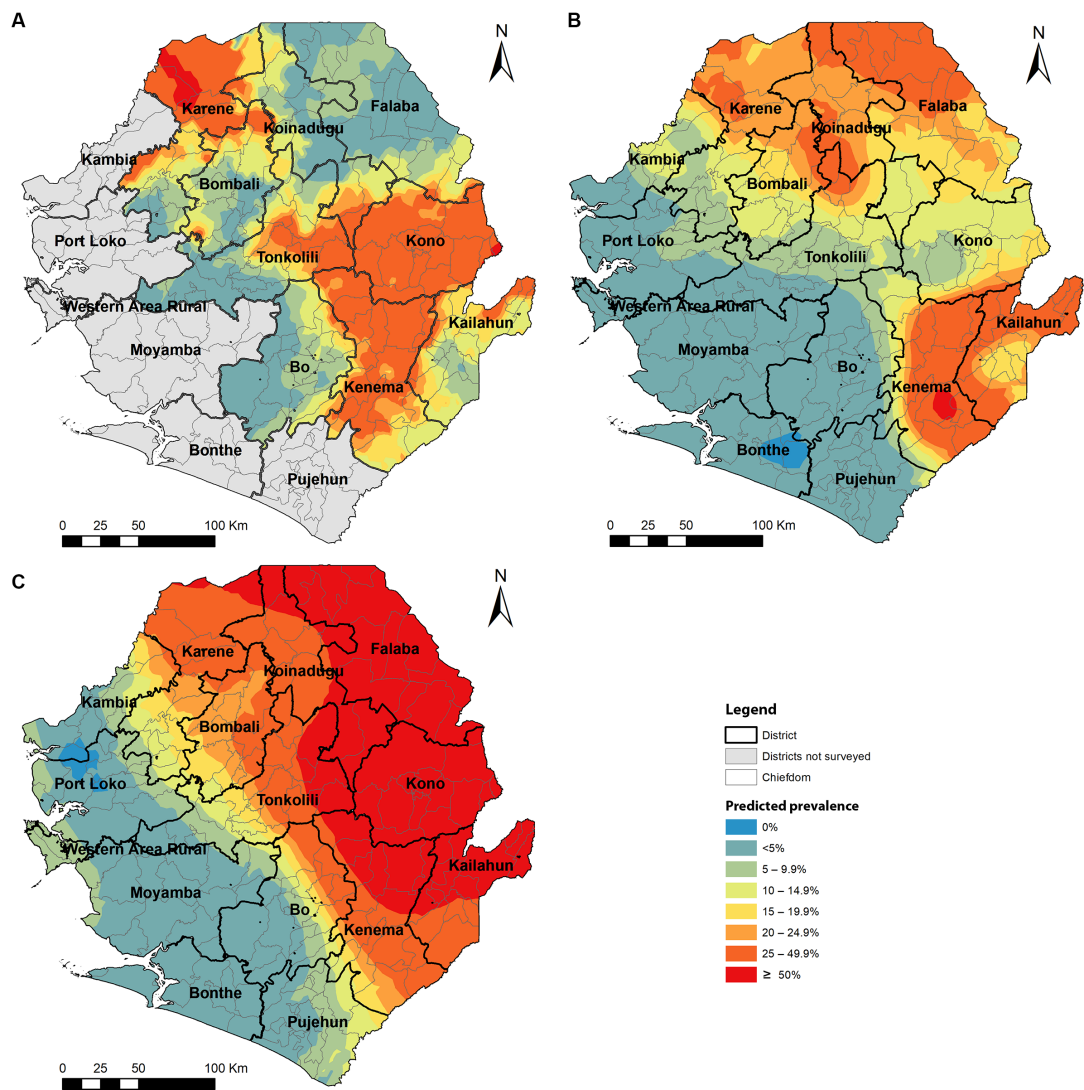
Spatially smoothed contour maps of predicted schistosomiasis prevalence in 2022 **(A)**, 2016 **(B)**, and of *S. mansoni* only at baseline in 2008 **(C)** in Sierra Leone. The prevalence of any species infection was not available in 2008 as the survey for *S. haematobium* was conducted separately, and the baseline *S. mansoni* prevalence was used as a proxy.

Among 125 chiefdoms surveyed, the mean prevalence of any species infection was <10% in 65 chiefdoms, 10–49.9% in 47 chiefdoms, and ≥ 50% in 13 chiefdoms, ranging from 0 to 91.7%. Spatial analysis showed that in 2022, chiefdoms in Kono, Kenema, and northern Karene had homogenous distribution of high prevalence of any species (>20%) across almost all the chiefdoms in these areas, while most chiefdoms in Bombali, Koinadugu, Falaba, Kailahun, and Tonkolili showed relatively low prevalence (Figure 4A). Compared with the predicted prevalence in 2016 (Figure 4B), there was a noticeable shift in prevalence distribution, i.e., reduction in chiefdoms in Falaba and Koinadugu but increase in chiefdoms in Kono and part of Tonkolili. Compared with the predicted prevalence at baseline (Figure 4C), there was a major reduction and shift in distribution of schistosomiasis prevalence across nine districts.

Overall, the prevalence among boys and girls was 17.6% (95% CI: 16.1–19.2%) and 17.9% (95% CI: 16.3–19.5%), respectively (Table 2). The prevalence was 18.1% (95% CI: 16.6–19.7%) for younger children (5–9 years old) and 17.2% (95% CI: 15.7–18.7%) for older children (≥10 years old), respectively (Table 2). There was no statistically significant difference of overall prevalence between either sex or age groups.

**Table 2:** Prevalence of schistosomiasis in school-aged children by age group and sex in 2022 in Sierra Leone.

	Number of children examined (proportion)	Prevalence (%) of any species infection	*p*-value
Overall	4,736	17.7 (16.7–18.8)	
Age group			
5–9 years^*^	2,364 (50.0%)	18.2 (16.6–19.7)	0.317
≥10 years	2,366 (50.0%)	17.2 (15.7–18.7)	
*Sex*			
Female	2,329 (49.1%)	17.9 (16.3–19.5)	0.317
Male	2,407 (50.8%)	17.6 (16.1–19.2)	

^*^Six children aged 4 years and younger are excluded.

### 3.3 Intensity of infection

The arithmetic mean egg counts for *S. mansoni* and *S. haematobium* (urine filtration) were 29.8 epg (95% CI: 24.6–35.0 epg) and 0.16 eggs/10 mL (95% CI: 0.08–0.23 eggs/10 mL) among SAC examined, respectively (Table 3). The prevalence of heavy infections for *S. mansoni* (69 of 4,736) and *S. haematobium* (1 of 4,618) in children tested was 1.5% (95% CI: 1.1–1.9%) and 0.02% (95% CI: 0.0–0.14%), respectively.

**Table 3:** Mean egg counts of schistosome infection in school-aged children in 2022 in Sierra Leone.

	Number of school children examined with stool samples	Number of stool samples tested positive	*S. mansoni* arithmetic mean egg count (epg) (95% CI)	Number of school children examined with urine samples	Number of urine samples tested positive by filtration^*^	*S. haematobium* arithmetic mean egg count (eggs/10 mL) (95% CI)	Number of heavy intensity infection of any species	Prevalence (%) of heavy intensity infection of any species (95% CI)
Overall	4,736	774	29.8 (24.6–35.0)	4,618	81	0.16 (0.08–0.23)	70	1.5 (1.2–1.9)
*District*								
Bo	432	14	27.1 (1.4–52.8)	429	0	0	4	0.9 (0.3–2.5)
Bombali	418	16	2.6 (1.0–4.2)	418	22	0.55 (0–1.31)	1	0.2 (0–1.5)
Falaba	330	4	0.6 (0–1.2)	329	13	0.12 (0.06–0.19)	0	0 (0–1.4)
Kailahun	670	96	4.7 (3.6–5.8)	645	0	0	0	0 (0–0.7)
Karene	368	88	124.2 (80.9–167.6)	366	8	0.08 (0.02–0.15)	36	9.8 (7.0–13.4)
Kenema	685	180	22.3 (18.6–26.0)	629	30	0.61 (0.38–0.83)	1	0.1 (0–0.9)
Koinadugu	407	12	0.4 (0.2–0.7)	406	0	0	0	0 (0–1.2)
Kono	737	257	61.7 (43.1–80.2)	733	3	0.02 (0–0.04)	16	2.2 (1.3–3.6)
Tonkolili	689	107	26.6 (20.0–33.2)	663	5	0.03 (0.0–0.05)	12	1.7 (0.9–3.1)
*Age groups*								
5–9 years	2,364	401	28 (21.9–34.0)	2,299	42	0.20 (0.05–0.34)	32	1.4 (0.9–1.9)
≥10 years	2,366	373	31.7 (23.1–40.2)	2,314	39	0.11 (0.07–0.15)	38	1.6 (1.2–2.2)
*Sex*								
Female	2,329	382	29.1 (22.4–35.8)	2,266	38	0.18 (0.04–0.33)	35	1.5 (1.1–2.1)
Male	2,407	392	30.5 (22.5–38.4)	2,352	43	0.13 (0.08–0.18)	35	1.5 (1.0–2.0)

^*^There were 21 Hemastix-negative urine samples which were tested by filtration, and the results were included in the urine results.

The arithmetic mean egg counts ranged from 0 to 124.2 epg for *S. mansoni* and from 0.0 to 0.61 eggs/10 mL for *S. haematobium* by district, with Karene having the heaviest infection (124.2 epg). Among chiefdoms, the arithmetic mean egg counts ranged from 0.0 to 629.5 epg for *S. mansoni* and from 0.0 to 6.67 eggs/10 mL for *S. haematobium* and 9 chiefdoms in Karene, Kono, Tonkolili, and Bo city having mean egg counts over 100 epg, with highest in Tambaka Yombanyie (629.5 epg) and Sella Limba (356.7 epg). Heavy intensity infections were found in 16 chiefdoms with the prevalence of heavy infection ranging from 1.4 to 45.8%. There were two sites in Karene district (one in Sella Limba and one in Tambaka Yombanyie) having mean egg counts for *S. mansoni*, 611.0 epg and 629.50 epg with prevalence of heavy infection of 70.8 and 45.8%, respectively. There was only one case of heavy mean egg count for *S. haematobium* found in Bo city.

The overall arithmetic mean egg count of *S. mansoni* infection in boys and girls was similar to 30.5 epg (95% CI: 22.5–38.4 epg) and 29.1 epg (95% CI: 22.4–35.8 epg), respectively. The overall arithmetic mean egg count of *S. mansoni* infection in older children (≥10 years) and younger children (5–9 years) was also similar with 31.7 epg (95% CI: 23.1–40.2 epg) and 28 epg (95% CI: 21.9–34.0 epg), respectively.

### 3.4 Prevalence and water, sanitation, and hygiene

The proportion of children attending schools with a basic drinking water service was 47.6%, limited service was 3.5%, and those without water service was 48.9% (Table 4). There was a significant difference in schistosomiasis prevalence for children by access to drinking water at school, with increased risk of infection in schools with no or limited access to drinking water (*p* < 0.001). In total, 57.9% of children had no access to latrines at school. There was a significant difference in the number of children who tested positive for schistosomiasis with a greater prevalence associated with basic sanitation services in the schools. In total, 39.8% of children had no access to handwashing facilities in school. There was no significant difference in schistosomiasis prevalence by access to handwashing facilities in school. As shown in Table 4, the proportion of students who tested positive for schistosomiasis was significantly higher in communities with no freshwater body (19.7%) when compared with children in communities where there is freshwater body (15.5%).

**Table 4:** Relationship between school water, sanitation, and hygiene indicators and prevalence in school-aged children in 2022 in Sierra Leone.

WASH indicators	Number of children tested (*n* = 4,664)	Prevalence (%) of any SCH (95% CI)	
Access to drinking water			
Basic	2,218 (47.6%)	15.6% (14.2–17.2)	<0.001
Limited	164 (3.5%)	19.5% (13.9–26.6)	
No service	2,282 (48.9%)	19.9% (18.3–21.7)	
Access to sanitation service			
Basic	1795 (38.5%)	21.6% (19.7–23.5)	<0.001
Limited	167 (3.6%)	13.2% (8.6–19.5)	
No service	2,702 (57.9%)	15.7% (14.4–17.2)	
Access to hygiene service			
Basic	1,577 (33.8%)	17.6% (15.8–19.6)	0.885
Limited	1,232 (26.4%)	17.7% (15.6–20.0)	
No service	1855 (39.8%)	18.2% (16.5–20.1)	
Communities with freshwater body used for recreational purposes			
No freshwater body	2,614 (56.1%)	19.7% (18.2–21.3)	<0.001
Freshwater body	2050 (44.0%)	15.5% (14.0–17.2)	

## 4 Discussion

Overall, the results of the recent impact assessment showed that the prevalence of any schistosomiasis has decreased since the start of the program, with prevalence of *S. mansoni* infection among SAC at 16.3% and *S. haematobium* infection at 2.0% in 2022. Despite this progress over time, the prevalence has remained at a relatively high level but importantly with few heavy infections (1.5% *S. mansoni* and 0.02% *S. haematobium*). There was no statistical comparison of the 2022 data with the 2008 baseline or 2016 assessment results as the sampling method was different. However, the results did show continued downward trend in overall prevalence from 2016 and massive reduction from 2008. Before 2016, national treatment strategy targeted both SAC and at-risk adults using the procured praziquantel due to the high baseline prevalence in these districts (13), as recommended in the WHO 2006 recommendations (4). From 2017, the national treatment strategy switched to treating SAC only due to the reduction in prevalence and the limited availability of the donated praziquantel through WHO. It was suggested that community-wide treatment including the adult population would be needed to achieve elimination of schistosomiasis as a public health problem in high prevalence settings (19–22). Sierra Leone had high schistosomiasis prevalence in several districts at baseline (8, 9). A community-based survey conducted in Bo district in 2018, 1 year after stopping treatment of the at-risk adult population, showed that adults had similar prevalence to that in SAC (16% in adults vs. 13.9% in SAC) (23). The shift in the national treatment strategy by stopping treatment of the at-risk adult population may have contributed to the relatively high prevalence in 2022 as the adult population could continue to serve as infection reservoir for transmission in the communities (22, 24). This suggests that stopping treatment in adults too soon, i.e., as soon as the prevalence in SAC becomes lower, may be risky. It is therefore important to include the at-risk adults for treatment, particularly, in chiefdoms with high prevalence in Kono, Kenema, Karene, and Tonkolili districts as discussed below. Pre-school children (aged 24–59 months) in these areas should also be considered for treatment when pediatric praziquantel becomes available as schistosomiasis prevalence in this group of children was high at baseline (25).

Most (52%) of chiefdoms showed low schistosomiasis prevalence (<10%), but there were 13 (10.4%) chiefdoms showing high prevalence (≥50%). These high prevalence chiefdoms are located mainly in Kono, Kenema, Karene, and Tonkolili districts, where the average district prevalence ranged from 16.1 to 35.4% in 2022. Kono and Kenema showed high prevalence in almost all chiefdoms across the two districts. In fact, Kono and Tonkolili districts showed increased prevalence from the 2016 survey, despite the major reduction in prevalence from the baseline in 2008 (Figure 4). It is noted that in Karene districts, two chiefdoms (Sella Limba and Tambaka Yombanyie) had sites with very high prevalence (>90%) and very high prevalence of heavy infection (70.8 and 45.8%, respectively). Persistent high prevalence in these districts may also reflect potential issues in the qualities of MDA implementation in these districts, apart from the stopping of adult treatment discussed above. In contrast, Falaba and Koinadugu districts had high prevalence (21.6–82.1%) at baseline in 2008 (8), and the average prevalence in 2022 was below 5%. This showed a massive progress in eliminating schistosomiasis in these districts. However, such comparison in data should be interpreted with caution. Different site selection strategies were used in these surveys: systematic cluster sampling in 2022 versus purposeful site selection previously (8, 13). Systematic sampling led to surveying different villages from those surveyed in previous surveys. Schistosomiasis is a focal disease, with prevalence varying widely among communities (1). Villages with high schistosomiasis transmission may have been missed, and prevalence may have been underestimated. After many years of MDA intervention, it is a major concern that such a high prevalence was found in many areas, particularly over 50% prevalence in 13 chiefdoms and mean egg count of over 100 epg in 9 chiefdoms, the schistosomiasis “hotspots”. It is recommended that the national program conducts a national review of the treatment data at a lower (e.g., community) level to identify potential issues in MDA implementation and factors causing high level transmission and design appropriate implementation strategies to ensure that the required geographical and epidemiological coverage are achieved. The national program should also consider conducting community-wide MDA in high-prevalence chiefdoms.

Schistosomiasis is transmitted by intermediate host snails in areas with water bodies where such snails are present. MDA alone is not able to achieve elimination of schistosomiasis in African context (26, 27), and the WHO recommends focal snail control to help reduce transmission (5, 28). The current results showed that MDA in SAC alone, coupled with missed rounds of MDA in 2018 and 2020, in Sierra Leone was not enough to reduce schistosomiasis prevalence to a low level in those high-endemicity chiefdoms. Apart from community-wide MDA discussed above, some other interventions may be needed, such as snail control and WASH. However, due to the lack of funding for snail control, it is unrealistic to conduct any snail control activities in schistosomiasis program in Sierra Leone. Access to safe water and adequate sanitation are associated with less schistosomiasis (29, 30). The current survey showed mixed relationship of schistosomiasis prevalence in school children with access to safe drinking water, access to sanitation facilities (toilets), and access to handwashing facilities in schools and having no freshwater bodies in communities seemed to have more schistosome infections. School WASH is less effective in controlling *S. mansoni* than household WASH, and sanitation may not be effective in controlling *S. haematobium* anywhere (31). This is due to the human habit of defecation and urination, and schools are rare places of schistosome transmission. Our results in schools may not reflect the true relationship of WASH with schistosomiasis in communities. The national program should collaborate with the WASH sector to assess and implement WASH activities in communities for households to maximize the effect of WASH on schistosomiasis elimination in Sierra Leone.

Based on the 2016 survey data, Sierra Leone transitioned district-level MDA to sub-district/chiefdom-level MDA. However, in the 2016 survey, only 5–6 sites per district were purposefully surveyed in known transmission areas in these nine districts (13). Surveys with such a site selection may not provide accurate estimate of the prevalence in a given district and may lead to mis- (over- or under-) estimation of prevalence and hence over/under treatment. Following the initial mapping surveys in 2008 (8), the Sierra Leone national program had to conduct a supplementary survey to have more survey sites in each district to facilitate the program decision on treatment strategy for each district (9). In the current impact assessment survey, we employed a population-based cluster sampling survey strategy with an average of 20 sites per district to improve and refine the chiefdom-level treatment strategies. It has been suggested that a precision mapping should be conducted to provide more granular data to facilitate better decision-making for transition from district-level MDA to sub-district level MDA (32). Such precision surveys have been conducted by others (33, 34). More recently, the Schistosomiasis Oversampling Survey (SOS) studies recommended conducting two-staged impact assessments with the first stage using systematic cluster survey at district level and the second stage using purposeful precision survey if the first stage survey shows heterogeneous disease distribution (35). The survey site selection strategy used in our survey in 2022 was similar to that recommended by the SOS studies. The survey results in 2022 in conjunction with the historical prevalence data facilitated the national program to make clear MDA decisions in 78 (60%) of 130 chiefdoms (there are more chiefdoms now due to new chiefdoms created) in nine districts, particularly in Kono with homogeneously high prevalence across all district chiefdoms. There were 29 chiefdoms having insufficient data for clear decision due to heterogeneous prevalence distribution or insufficient number of villages surveyed. Further chiefdom-level assessment in these 29 chiefdoms is to be conducted in additional villages, and more data points in these chiefdoms will facilitate better decision-making. In addition, as discussed above, although 23 chiefdoms in Falaba and Koinadugu districts had almost homogeneously low prevalence results, it is suggested that these two districts should also be included in the further chiefdom-level assessment to verify the current prevalence situation.

There are certain limitations in this survey. First, the survey was school-based and only school children present in the school on the survey day were surveyed. School children who were absent or out-of-school children in communities were not surveyed. This may cause slight misrepresentation of true community prevalence. Second, the Kato-Katz technique was used in this survey. Although two slides are much more sensitive than one slide in Kato-Katz, it is still insensitive for low intensity infections (36–38), particularly in settings after years of MDA intervention. Therefore, the results may have still underestimated the true prevalence. The same may be true for Hemastix which may not be a stable proxy for estimating *S. haematobium* prevalence in low prevalence setting (39), such as in Sierra Leone. However, these should not affect program decision as decision for each chiefdom would be made based on holistic review of prevalence trends from all available data from baseline to current situation.

## 5 Conclusion

Impact assessment of schistosomiasis program using cluster sampling strategy was conducted in 2022 in Sierra Leone and provided sufficient data for sub-district level MDA decision for most (60%) of 130 chiefdoms in nine districts. Data gaps are identified in some chiefdoms, and further chiefdom-level assessment is needed in these chiefdoms to refine the MDA decision. The results showed that Sierra Leone has made significant progress in reducing schistosomiasis prevalence across the country after a decade of MDA intervention. However, there remains high prevalence in some hotspot chiefdoms in Kono, Karene, Kenema, and Tonkolili districts. The national program should conduct a program review on treatment data at chiefdom or community level, particularly in hotspot chiefdoms, to identify potential issues and improve MDA quality, accordingly, conduct chiefdom-level assessment in chiefdoms with data gaps to refine treatment strategies, consider increasing treatment frequency and expanding targeted treatment to community-wide treatment in hotspot chiefdoms or communities, and consider other control measures such as focal snail control if feasible and WASH in communities.

## Data availability statement

All data generated or analyzed during this study are included in this published article. The dataset analyzed is available from the National Neglected Tropical Disease Program on reasonable request and can be made available with permission from the Ministry of Health and Sanitation, Sierra Leone.

## Ethics statement

The schistosomiasis impact assessment was part of the monitoring and evaluation activities of the national NTD elimination program and was conducted by the NTDP of the Ministry of Health and Sanitation (MOHS) Sierra Leone per WHO recommendations. Ethical approval for the survey was obtained from the Ethics Committee of the MoHS, Sierra Leone. Community informed consent was obtained following discussions with District Medical Officers, chiefdom school inspectors, head teachers and community-teachers associations. All communities included in the survey were sensitized prior to sample collection and verbal consent was obtained from parents during this meeting. Written consent was also obtained from the school authorities and the village heads on behalf of the pupils recruited in the survey. Children recruited were sensitized on the purpose of the activity prior to sample collection and were free to drop out of the process at any point. Each selected child who agreed to participate was registered and designated an identification code. The dataset was kept in the national NTD database at MoHS. No personal information can be revealed in the final survey report or upon publication of this paper.

## Author contributions

IK-L: Project administration, Supervision, Writing – review & editing. VR-S: Data curation, Investigation, Project administration, Writing – review & editing. AC: Investigation, Project administration, Supervision, Writing – review & editing. EC: Data curation, Formal analysis, Writing – review & editing, Validation, Visualization. MB: Investigation, Project administration, Writing – review & editing. AK: Data curation, Supervision, Writing – review & editing. UK: Investigation, Supervision, Writing – review & editing. LK: Data curation, Formal analysis, Writing – review & editing. SJ: Project administration, Writing – review & editing. PH: Project administration, Writing – review & editing. SR: Project administration, Writing – review & editing. DE: Methodology, Writing – review & editing, Conceptualization. AW: Writing – review & editing, Project administration. AP: Conceptualization, Methodology, Writing – review & editing. YZ: Conceptualization, Formal analysis, Methodology, Supervision, Visualization, Writing – original draft, Writing – review & editing.
